# The Hexamer Structure of the Rift Valley Fever Virus Nucleoprotein Suggests a Mechanism for its Assembly into Ribonucleoprotein Complexes

**DOI:** 10.1371/journal.ppat.1002030

**Published:** 2011-05-12

**Authors:** François Ferron, Zongli Li, Eric I. Danek, Dahai Luo, Yeehwa Wong, Bruno Coutard, Violaine Lantez, Rémi Charrel, Bruno Canard, Thomas Walz, Julien Lescar

**Affiliations:** 1 Architecture et Fonction des Macromolécules Biologiques, Marseille, France; 2 Harvard Medical School, Department of Cell Biology, Boston, Massachusetts, United States of America; 3 Howard Hughes Medical Institute, Harvard Medical School, Boston, Massachusetts, United States of America; 4 Nanyang Technological University, School of Biological Sciences, Singapore; 5 Unité des Virus Emergents, Université Aix-Marseille II et Institut de Recherche pour le Développement, Marseille, France; Institut Pasteur, France

## Abstract

Rift Valley fever virus (RVFV), a *Phlebovirus* with a genome consisting of three single-stranded RNA segments, is spread by infected mosquitoes and causes large viral outbreaks in Africa. RVFV encodes a nucleoprotein (N) that encapsidates the viral RNA. The N protein is the major component of the ribonucleoprotein complex and is also required for genomic RNA replication and transcription by the viral polymerase. Here we present the 1.6 Å crystal structure of the RVFV N protein in hexameric form. The ring-shaped hexamers form a functional RNA binding site, as assessed by mutagenesis experiments. Electron microscopy (EM) demonstrates that N in complex with RNA also forms rings in solution, and a single-particle EM reconstruction of a hexameric N-RNA complex is consistent with the crystallographic N hexamers. The ring-like organization of the hexamers in the crystal is stabilized by circular interactions of the N terminus of RVFV N, which forms an extended arm that binds to a hydrophobic pocket in the core domain of an adjacent subunit. The conformation of the N-terminal arm differs from that seen in a previous crystal structure of RVFV, in which it was bound to the hydrophobic pocket in its own core domain. The switch from an intra- to an inter-molecular interaction mode of the N-terminal arm may be a general principle that underlies multimerization and RNA encapsidation by N proteins from *Bunyaviridae*. Furthermore, slight structural adjustments of the N-terminal arm would allow RVFV N to form smaller or larger ring-shaped oligomers and potentially even a multimer with a super-helical subunit arrangement. Thus, the interaction mode between subunits seen in the crystal structure would allow the formation of filamentous ribonucleocapsids *in vivo.* Both the RNA binding cleft and the multimerization site of the N protein are promising targets for the development of antiviral drugs.

## Author Summary

The Rift Valley fever virus (RVFV), a negative strand RNA virus spread by infected mosquitoes, affects livestock and humans who can develop a severe disease. We studied the structure of its nucleoprotein (N), which forms a filamentous coat that protects the viral RNA genome and is also required for RNA replication and transcription by the polymerase of the virus. We report the structure of the RVFV N protein at 1.6 Å resolution, which reveals hexameric rings with an external diameter of 100 Å that are formed by exchanges of N-terminal arms between the nearest neighbors. Electron microscopy of recombinant protein in complex with RNA shows that N also forms rings in solution. A reconstruction of the hexameric ring at 25 Å resolution is consistent with the hexamer structure determined by crystallography. We propose that slight structural variations would suffice to convert a ring-shaped oligomer into subunits with a super-helical arrangement and that this mode of protein-protein association forms the basis for the formation of filamentous ribonucleocapsids by this virus family. Both the RNA binding cleft and the multimerization site of the N protein can be targeted for the development of drugs against RVFV.

## Introduction

The *Bunyaviridae* family comprises more than 330 viruses that affect vertebrates and plants. La Crosse virus, a member of the *Orthobunyavirus* genus, causes pediatric viral encephalitis in North America. The *Bunyaviridae* family also includes several other emerging human pathogens, such as the Hantaan and Sin Nombre viruses (genus *Hantavirus*) and the Crimean-Congo hemorrhagic fever virus (genus *Nairovirus*). Viruses of the *Tospovirus* genus infect plants [1]. *Bunyaviridae* have either arthropods- or rodent-borne vectors and are amplified by vertebrate hosts. The Rift Valley fever virus (RVFV), a *Phlebovirus* within the *Bunyaviridae* family, is transmitted by *Aedes* and *Culex* mosquitoes and is a medically and agriculturally important cause of epizootics in Africa. Although this virus primarily affects livestock, humans can be infected as well, and infections can lead to several syndromes ranging from a febrile illness to blindness, encephalitis and lethal hemorrhagic fever. The virus is currently found in the sub-Saharan area, as well as in Egypt, Yemen, Saudi-Arabia, Mayotte and Madagascar [2]. The continuing geographical expansion of RVFV draws concern for Europe, where the virus is considered to be an emerging threat [3], [4]. Current vaccines to prevent RVFV epizootics are only partially attenuated, expensive and only induce short-lived immunity [5]. No specific drugs are available to cure an infection, and preventive efforts to avoid new outbreaks are mostly based on weather monitoring [6].

The genome of RVFV consists of three single-stranded RNA segments of either negative or ambisense polarity designated as L (6,404 nucleotides [nt]), M (3,885 nt), and S (1,690 nt). Within each of these three segments, coding regions are flanked at their 5′ and 3′ termini by non-translated regions that comprise two stretches of complementary nucleotides, leading to the formation of RNA panhandle structures [7]. The L and M segments are of negative polarity, while S has ambisense polarity, encoding the nucleoprotein (N) in antisense and the non-structural protein NSs in sense orientation. The L segment expresses a multifunctional protein that comprises an N-terminal endonuclease [8] and a large RNA-dependent RNA polymerase domain [1]. The M segment codes for glycoproteins G_N_ and G_C_ that are inserted in the virus lipid envelope and are responsible for cell tropism and membrane fusion. The endodomain of G_N_ interacts with N, and this interaction is critical for genome packaging into infectious virus particles.

As in other negative-stranded viruses, the genomic RNA (vRNA) in RVFV is packaged with two virally expressed proteins, N and L, into a ribonucleoprotein (RNP) complex that is competent for (+)RNA synthesis and transcription. Contrary to RNPs of Mononegavirales, RVFV N does not assemble into a tube-like structure [9]–[14] but rather forms a flexible serpentine-like structure [15]. The precise organization of RNA, N and L in this macrostructure is unknown. In addition to its critical role in protecting the vRNA and the antigenome (cRNA), the N protein also plays an active role in RNA transcription and replication [1], as well as in virion assembly [16]. Biochemical studies have shown that RVFV N forms dimers through aromatic residues located in the N terminus of the protein [17]. Recently, a crystal structure was reported for the RVFV N protein [15], which revealed the basic fold of the protein, but raised a number of questions. For example, the crystal structure provided little insight into the mechanism of N multimerization into an RNP complex, and it was unclear how to relate the crystal structure to EM images of N polymers. Furthermore, the RNA binding site identified in the crystal structure of RVFV N differed from that seen in other viral N proteins. Here, we present the crystal structure of RVFV N forming a hexameric ring. The structure reveals the likely binding site for vRNA, and comparison with the previous crystal structure of RVFV N allows us to speculate on the mechanism that underlies the multimerization of N and its encapsidation of viral RNA.

## Results

### Recombinant N protein forms oligomers and can bind RNA

**Figure 1:**
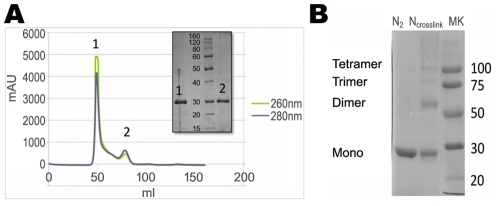
Oligomeric state of recombinant N protein. **(A)** Chromatogram of N run on an S200 size exclusion column. The grey line shows the absorbance at 280 nm, and the green line the absorbance at 260 nm. The inset shows a 12.5% SDS-PAGE gel of peaks 1 (N_1_) and 2 (N_2_). **(B)** A 12.5% SDS-PAGE gel showing the presence of dimers, trimers and tetramers after cross-linking fraction N_2_ with 0.05% glutaraldehyde for one hour at room temperature.

To produce sufficient amounts of protein for structural studies in the absence of other viral proteins, we expressed RVFV N in *E. coli* with an N-terminal cleavable thioredoxin tag and purified it under non-denaturing conditions to preserve its structural integrity. The final gel filtration column showed two peaks, denoted as N_1_ and N_2_ (**Figure 1A**). SDS-PAGE analysis revealed that both peaks contained a protein of the size expected for N (27 kDa), suggesting that N was the only protein present and ruling out protein contaminants that could have influenced the oligomeric state of N (**Figure 1A**, inset). The position of peak N_1_ corresponds to a protein species with an apparent molecular mass of 300 kDa, suggesting that N formed higher-order oligomers. The position of peak N_2_ corresponds to a protein species with an apparent molecular mass of 94 kDa and would thus suggest the presence of smaller oligomers. This notion was confirmed by cross-linking experiments that indicated the presence of dimers, trimers and tetramers in fraction N_2_ (**Figure 1B**). We also measured the OD_260nm_/OD_280nm_ ratio for the two peak fractions to test for the presence of bound nucleic acids. Peak N_1_ had an OD_260nm_/OD_280nm_ ratio of 1.19, clearly indicating that the higher-order N oligomers co-eluted with nucleic acids [18], presumably RNA from the expression host. In contrast, the OD_260nm_/OD_280nm_ ratio of the N_2_ peak was 0.72, showing that this fraction contained much less RNA than fraction N_1_ [18]. The variability in the oligomeric state of N expressed in *E. coli* is consistent with previous studies that used either N purified from infected cells or recombinant N expressed in insects cells, although in the latter case multimers with a higher MW were observed [16], [17].

We next used surface plasmon resonance experiments to test whether the recombinant protein in fraction N_2_ retained its capacity for non-specific RNA binding. We measured the interaction of N with a 20-nucleotides-long RNA, and determined that the Kd of N for RNA is 3.8 µM (**Figure S1**). This result demonstrated that the recombinant N protein present in peak N_2_ can still bind RNA and therefore presumably has the native fold. We therefore used this protein for 3D crystallization and structure determination.

### Structure determination, crystal packing and structure of the RVFV N protein

Using the N_2_ fraction, crystals were obtained in the P6 space group with unit cell parameters of a = b = 180.9 Å and c = 47.4 Å. The selenomethionyl protein crystallized in the same space group with similar unit cell parameters, a = b = 175.5 Å and c = 47.4 Å (**Table 1**). The structure was determined using the SAD technique with data recorded at the Se absorption edge from crystals of the selenomethionyl protein that diffracted to 2.3 Å resolution. The structure was subsequently refined using a native data set that extended to 1.6 Å resolution (**Table 1**).

**Table 1:** Data collection and refinement statistics.

Data collection and refinement statistics of RVFV nucleoprotein	Se-MET^a^	Native^b^
Instrument	ESRF - ID14–4	ESRF - ID14–4
Wavelength	0.9790	0,9770
Space group	P6	P6
Cell dimensions *a*, *b*, *c* (Å)/α, β, γ (°)	175.5, 175.5, 47.4/90, 90, 120	180.9, 180.9, 47.7/90, 90, 120
Resolution range (Å)	47.44– 2.30 (2.42–2.30)* &	47.1–1.6 (1.69 – 1.6)*
Total number of reflections	524146 (73999)*	575561 (39900)*
Number of unique reflections	37702 (5378)*	111024 (13148)*
Completeness (%)	99.6 (97.6)*	93.8 (76.5)*
*I*/Σ(*I)*	27.5 (15.6)* &	10.9 (2.0)*
R_sym_ **	0.072 (0.130)* &	0.084 (0.510)*
Multiplicity	13.9 (13.8)*	5.2 (3.0)*
**Refinement**		
R***	0.2015	0.2221
R_free_ *****	0,2505	0.2536
**No. atoms**		
Protein	5781	5901
Water	347	965
**B-Factors**		
Protein	14.14	19.16
Water	19.14	33.2
**R.m.s. deviations**		
Bonds lengths (Å)	0,0078	0.0061
Bonds angles (°)	1.023	0.883

a PDB code: 3OUO

b PDB code: 3OU9

& A resolution cutoff of 2.3 Å was chosen to retain only the most reliable data for phasing purposes.

*Values in parentheses give the values in the highest resolution shell (1.69–1.6 Å ).

**R_sym_ = Σ |I-<I>|/Σ I

***R = Σ||Fo|-|Fc||/Σ |Fo|

*****Rfree = R factor calculated using 5% of reflections not included in refinement.

**Figure 2:**
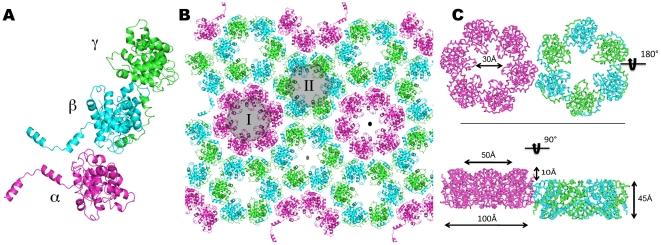
Hexamers formed by N in the crystal. **(A)** The three N monomers in the asymmetric unit are shown in pink, cyan and green and labeled α, β and γ, respectively. **(B)** Subunit packing in the crystal layer corresponding to the crystallographic [*a,b*] plane. The six copies of subunit α that surround the crystallographic 6-fold symmetry axis (black dot) form one hexamer (labeled I), and the three βγ dimers that surround the crystallographic 3-fold symmetry axis (grey dot) form a second hexamer (labeled II). **(C)** The dimensions of the hexameric ring are labeled. Hexamers I and II face in opposite directions and are offset by approximately 10 Å in the direction of the *c* axis.

The asymmetric unit contains three N molecules, labeled α, β and γ in **Figure 2A**, that form two distinct hexameric rings in the crystal, labeled I and II in **Figure 2B**. Hexamer I is formed by six copies of subunit α that surround the crystallographic 6-fold axis, whereas hexamer II is formed by three β,γ dimers that surround the crystallographic 3-fold axis (**Figure 2B**). The two sets of hexamers, which face in opposite directions and are offset by 10 Å in the direction of the crystallographic *c* axis (**Figure 2C**), form layers along the [*a,b*] plane of the crystal. Stacking of the layers in the crystal results in the formation of two sets of tubes, one set formed by hexamers I and the other by hexamers II, that both run along the crystallographic *c* axis but in opposite directions (**Figure S2**).

**Figure 3:**
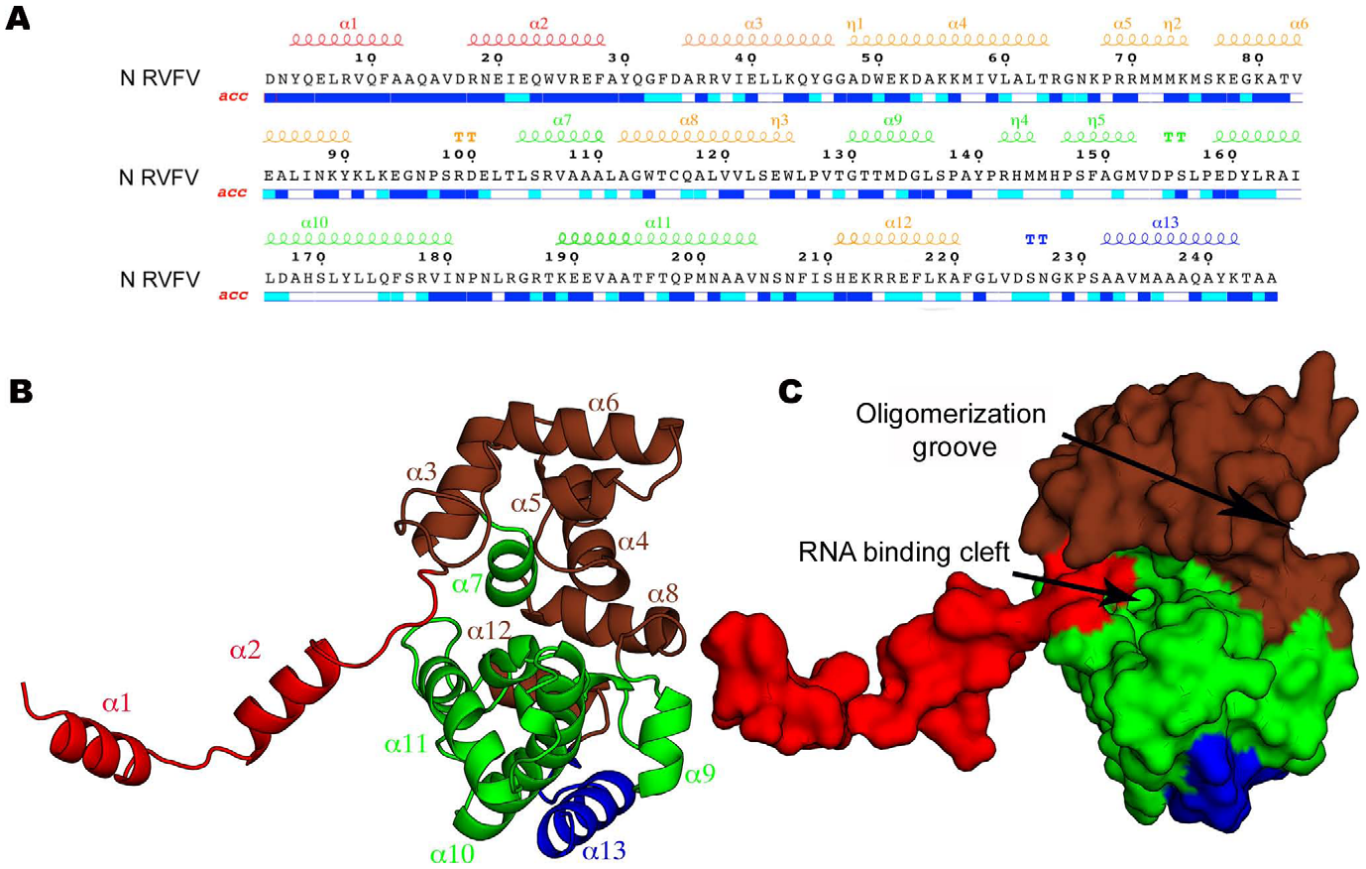
Structure of the N monomer. **(A)** Sequence of the RVFV N protein with secondary structure elements indicated above. The colors correspond to those used to color the different sub-domains in the crystal structure of N shown in panels B and C. **(B)** Ribbon representation of the crystal structure of the RVFV N protein showing that the N terminus forms an arm (red) that extends from the globular core domain (brown and green). The C terminus, which is not involved in RNA binding, is shown in blue. The α-helices are labeled. **(C)** View of the RVFV N protein in surface representation. The orientation and color code are the same as in panel B.

The crystal structure reveals that the N monomer consists of an orthogonal bundle of thirteen α-helices (**Figure 3**). The structure can be divided into three domains. Residues 1–32 form a flexible N-terminal arm containing two α-helical segments that extends away from the globular core of the protein. The globular core itself consists of two domains, one formed by six helices spread over residues 36–90, 110–122, 211–220 and the other one formed by four helices spread over residues 103–110 and 130–204 (**Figure 3**). The core domain in our structure is virtually identical to that in the previously reported crystal structure of N [15] with an rms deviation between the backbone atoms of the two structures of∼0.7 Å (**Figure S3B**). The position and conformation of the N-terminal arm, however, are very different in the two structures (**Figure S3**), a finding that will be discussed below. The fold of RVFV N is currently unique in the PDB, but considering the high level of conservation in their amino-acid sequences (average identity>30%) (**Figure S4**), other *Phlebovirus* N proteins are likely to adopt a similar fold.

### Multimerization of the N protein

**Figure 4:**
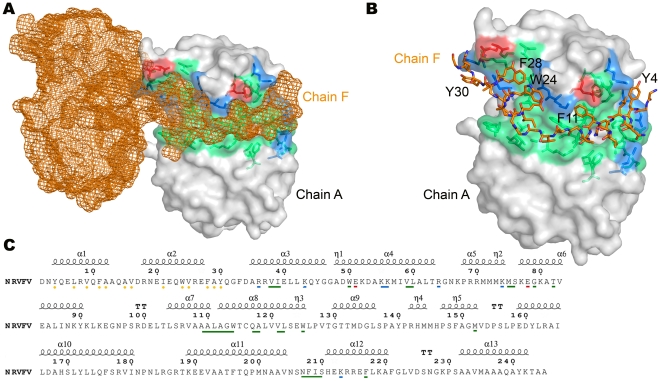
Interaction between adjacent N subunits in the hexamer. **(A)** Overview of how the N-terminal arm of one subunit, shown in orange mesh, fits into the hydrophobic pocket in the surface of the adjacent subunit, shown as grey surface with the hydrophobic pocket highlighted in green. **(B)** Magnified view of the interaction. The N-terminal arm is shown in ball-and-stick representation and the surface of the hydrophobic pocket is shown transparent to reveal details of the hydrophobic interactions. (**C**) Amino acid sequence of an RVFV N polypeptide, showing above the secondary structure elements derived from the crystal structure. Below the sequence, residues are labeled that are involved in inter-subunit interactions in the hexamer. The yellow dots indicate residues of the arm that interact with residues in the oligomerization groove. The colored bars indicate the character of the residues in the oligomerization groove: green, hydrophobic; blue, positively charged; red, negatively charged.

In our crystals, N forms ring-shaped hexamers (the subunits are denoted A to F as shown in **Figure S2**) that have a thickness of 45 Å, an external diameter of approximately 100 Å, and a central funnel-like aperture with a diameter that narrows from 50 to 30 Å (**Figure 2C**). Multimerization appears to be driven by the extended N-terminal arm, which wraps around the external surface of the globular core of the adjacent subunit, fitting snuggly into a hydrophobic groove and burying a surface of 1456 Å^2^ (**Figure 4A**). In particular, the aromatic rings of residues Y3, F11, W24, F28 and Y30 and the aliphatic side chains of residues L7, V9, V16, I21 and V25 project from the N-terminal arm and fill up the hydrophobic groove formed by regions 36–82, 108–126, and 207–210 of the core domain of the adjacent molecule (**Figure 4B** **/C**). This arm-core interaction is repeated in a directional manner, such that the arm of subunit A extends into the hydrophobic groove of subunit B, B into C, C into D, D into E, E into F and F into A, creating the hexameric rings seen in the crystal. This mode of multimerization is consistent with mutagenesis data that mapped the interacting domain of the *Phlebovirus* N protein to its N-terminal arm [17].

Hexamers I and II in the crystals of the native protein superimpose very well, but the two rings in the crystals of the selenomethionyl protein are slightly different (**Figure S5A**). While hexamer II formed by seleniated N is the same as the two hexamers formed by native N (**Figure S5B**), the subunits in hexamer I are more closely packed about the 6-fold symmetry axis. The domain of N that is near the center of the ring, comprising the loop connecting helices α10 and α11, occludes part of the central aperture, suggesting a twist in the assembly of the ring subunits (**Figure S5A**). Superimposition of native and seleniated hexamers I based on subunits A, creates an 11° deviation between the planes of the two rings (**Figure S5C**). Furthermore, comparison of the subunits in hexamers I formed by native and seleniated protein shows that the contraction of the ring is due to a lateral slippage between adjacent subunits (**Figure S6**). As a result of the slightly different subunit organization, the asymmetric unit is shorter in crystals of the seleniated protein and the length of the crystallographic *a* and *b* axes is decreased by about 5 Å (**Table 1**). The existence of two types of rings in the crystals demonstrates the natural ability of N to form oligomers with different subunit organizations, providing a basis for the formation of serpentine-like RNP structures.

### RNA binding

**Figure 5:**
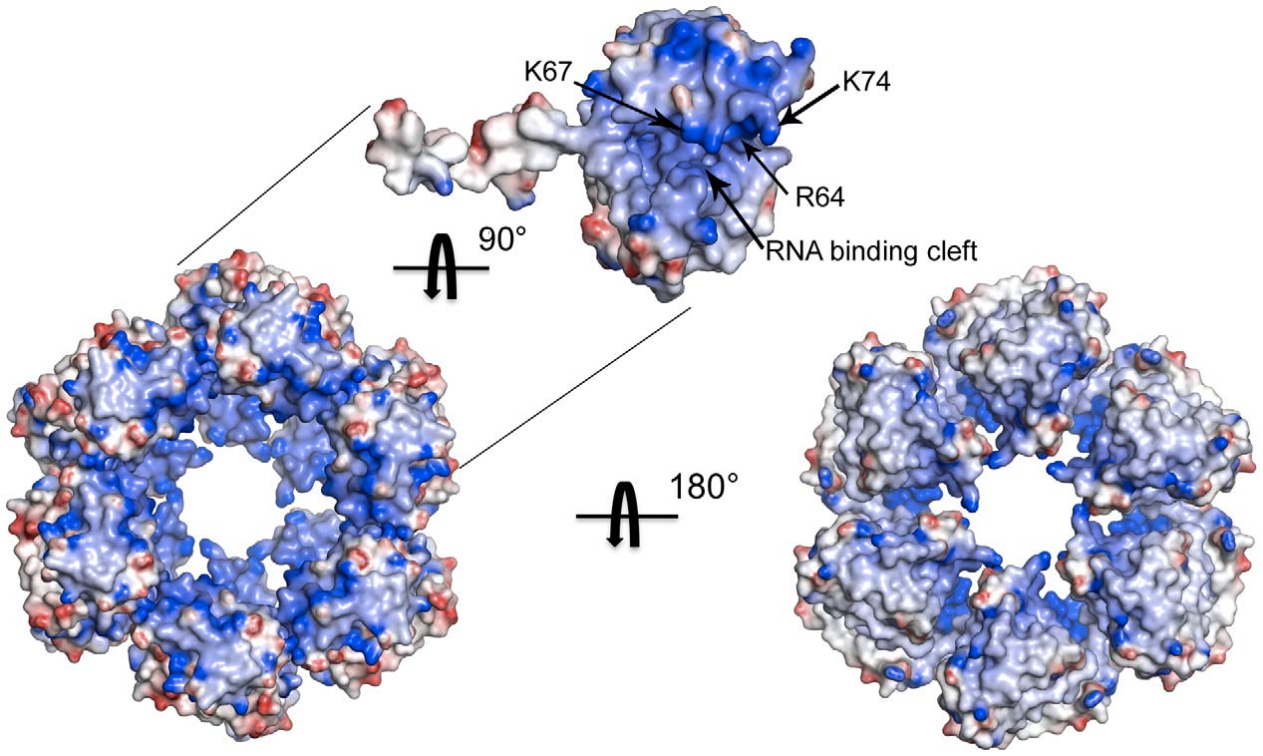
Electrostatic surface potential of the N hexamer. Mapping of the electrostatic surface potential, from −10 kT in red to+10 kT in blue, onto the surface of hexamer I formed by native N protein reveals a patch of positive charges in the inner part of the ring, which likely accommodates the vRNA. Key residues in the RNA binding site are labeled on the electrostatic surface of a single monomer.

The core of the N protein has a concave crescent shape and the relative orientation of its two domains is reminiscent of a head of pliers, suggestive of a role in grabbing genomic RNA (**Figure 3C**). This cleft is sandwiched between three helices on one side (α4, α5, α7) and two 3_10_-helices (η4, η5) followed by three α-helices on the other (α9, α10, α11), a fold in accordance with the “(5H+3H)” structural motif for RNA binding [19]. Furthermore, analysis of the electrostatic surface potential reveals a positively charged patch located within the inner part on one side of the hexamers (**Figure 5**). This patch includes residues R64, K67 and K74 that are evolutionary conserved across *Phleboviruses* (**Figure S4**). To test whether this positively charged patch indeed constitutes the RNA binding site, we expressed and purified a triple RVFV N mutant (R64D, K67D, K74D). The triple mutant eluted from the gel filtration column as a single peak, corresponding to N_2_ (**Figure S7A**). SDS-PAGE analysis revealed that the peak fractions contained a protein of the size expected for N (27 kDa) (**Figure S7A,** inset), and mass spectrometry confirmed the protein to be RVFV N. The OD_260nm_/OD_280nm_ ratio of the peak fraction was 0.52, indicating that this fraction contained only protein [20]. Binding studies using surface plasmon resonance spectroscopy with a 20-nucleotides-long RNA showed that the triple mutant lost its ability to bind RNA, supporting the notion that the positively charged patch serves as the RNA binding cleft (**Figure S7B**).

Taking as a guide the structure of the rabies virus N protein bound to single-stranded RNA (PDB code: 2GTT [11]), we could position an RNA molecule in the concave surface between the two core domains of the RVFV N protein, such that the RNA sugar phosphate backbone interacts with the positive charges in the basic cleft. The model of RVFV N protein bound to RNA further showed that each N subunit can accommodate approximately six RNA bases (**Figure S8**).

### Electron microscopy of N-RNA complexes

**Figure 6:**
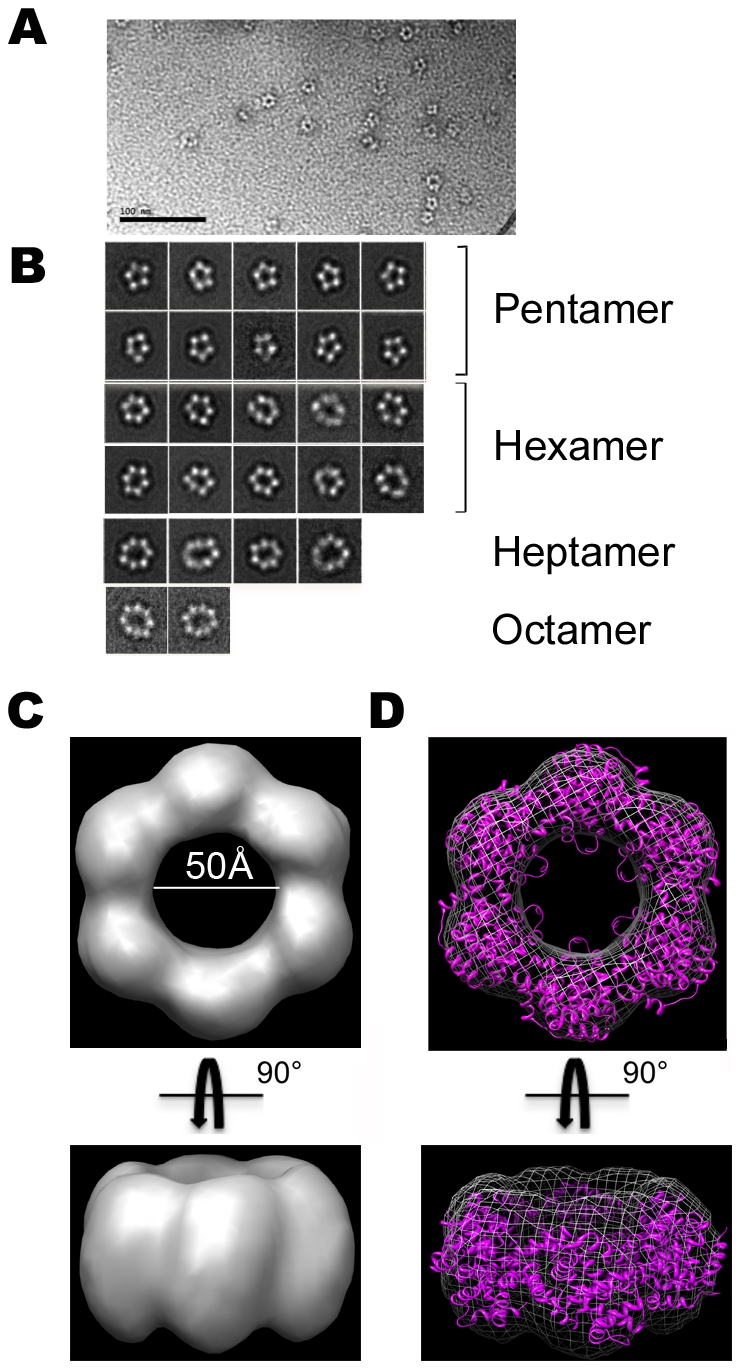
Electron microscopy of N-RNA complexes. **(A)** Representative electron micrograph of the N_1_ fraction in negative stain, revealing ring-shaped particles of different sizes. Scale bar is 100 nm. **(B)** Representative class averages of N-RNA rings. **(C)** 3D reconstruction of a hexameric N-RNA complex obtained with cryo-negatively stained samples. **(D)** Docking of the crystal structure of hexamer I formed by native N into the EM density map of the hexameric N-RNA complex.

The crystal structure showed that RVFV N forms hexameric rings. To assess whether N also forms hexamers in solution, we prepared negatively stained samples for analysis by electron microscopy (EM). EM images of fraction N_2_, which contained only protein and was used for 3D crystallization, did not show any ring-shaped complexes (data not shown), consistent with the SEC result that showed that this fraction contains only small oligomers. By contrast, EM images of fraction N_1_, which contained both protein and RNA, revealed distinct circular structures with diameters ranging from∼70 to 100 Å, which were stable over a period of one month (**Figure 6A** **)**. The images thus suggest that the formation of stable higher-order N oligomers requires the protein to associate with RNA and that the resulting higher-order oligomers have a ring-shaped structure.

To obtain a better understanding of the structure of N-RNA complexes, we calculated 3D reconstructions of the ring-shaped complexes seen in fraction N_1_. The small size of the complexes prevented us from using vitrified specimens for EM imaging, and we therefore prepared samples by cryo-negative staining. This specimen preparation method provides the high contrast of stain but minimizes the artifacts associated with conventional negative staining [21]. The N-RNA complexes adsorbed to the carbon support film preferentially with the flat side of the ring, making it necessary to use the random conical tilt approach to calculate 3D reconstructions [22]. We recorded a total of 30 image pairs at tilt angles of 50° and 0°, from which we selected 10,764 particle pairs. The particles from the images of the untilted specimen were classified into 100 classes, which revealed a variety of oligomers, ranging from tetramers to octamers (**Figures 6B** **and** **S9**). About 57% of all the particles were ring-shaped oligomers. The hexamer was the most abundant species with 24%, followed by the pentamer (22%), the heptamer (7%), and finally the octamer (4%). The averages of the various oligomers revealed a large variability in the ring shape, pointing to structural flexibility in the various N-RNA complexes. Because the hexamer was most prevalent and because N alone formed hexamers in the 3D crystals, we focused on calculating a 3D reconstruction of the hexameric N-RNA complex. We combined the particles from classes that produced the most similar averages (399 particles from 2 classes) and calculated a 3D density map using the particles selected from the images of the tilted specimen and the best 10% of particles selected from the untilted specimen. According to the Fourier shell correlation (FSC) = 0.5 criterion, the final density map had a resolution of 25 Å (**Figure S10**).

With a diameter of about 100 Å and a thickness of about 45 Å (**Figure 6C**), the EM density map of the N-RNA complex has virtually identical dimensions as the crystal structures of the N hexamer. Accordingly, the EM density map nicely accommodated the crystal structure of the hexamer, illustrating that the hexamers, and by extension also the other ring-shaped oligomers, are compatible with RNA binding (**Figure 6D**).

## Discussion

The N protein is the most abundant viral protein in the *Phlebovirus* virion and plays a key role in encasing vRNA in a protective coat. We have determined the crystal structure of RVFV N in a hexameric form, which shows largely the same fold that was previously seen in a crystal structure of monomeric N [15]. The two structures differ, however, in the position of the N-terminal arm. In the previous structure, the N-terminal arm packs closely against the core domain, while it extends away from it in our structure (**Figure S3**). Extension of the N-terminal arm is crucial for the oligomerization of N, as it mediates the interaction with the adjacent subunit in the crystallographic hexamer. We believe that the RVFV N hexamer is biologically relevant, because oligomers have been observed for many other N proteins [9], [10], [11], [12], [13], [14] and EM revealed that the RVFV N-RNA complex also forms ring-shaped oligomers in solution (**Figure 6**).

The different position of the N-terminal arm in our and the previous structure is intriguing as it may reflect the structural change that has to occur for N to multimerize, thus potentially providing a clue to the mechanism underlying the formation of a ribonucleocapsid. In the hexamer, the N-terminal arm lies in a hydrophobic pocket of the adjacent subunit, thus mediating an inter-molecular interaction (**Figure 4**). By contrast, in monomeric N, the N-terminal arm makes an intra-molecular interaction and binds to the same hydrophobic pocket but in its own core domain, burying a surface area of 1179 Å^2^ (**Figure S11**). The inter- and intra-molecular interactions with the N-terminal arm are mediated largely by the same residues of the core domain (**Figure 4C** and **Figure S11B**). Interestingly, the intra-molecular interaction of the N-terminal arm not only fills the hydrophobic pocket of its own core domain, thus preventing oligomerization, but also covers the RNA binding cleft, so that N in this conformation is incapable of binding RNA. For a monomer, the closed conformation is presumably more favorable, because it reduces the hydrophobic surfaces on both the N-terminal arm and oligomerization groove. In case the closed conformation is a “waiting” conformation before oligomerization; residues involved in the molecular interaction would have to compete for the oligomerization groove and expose the hydrophobic side of the arm (**Video S1**).

Our SEC analysis shows that peak N_2_, which lacks RNA, contains only small oligomers, suggesting that the inter-molecular interactions mediated by the N-terminal arm are not very strong on their own, potentially because the intra-molecular interactions outcompete the inter-molecular interactions, and thus do not support large oligomer formation. The weak interactions between N proteins would allow easy addition and removal of subunits. The fact that we see hexamers in our crystals may be explained by the high protein concentration used for crystallization trials that drives the small units of nucleoproteins to assemble into larger stable oligomers. In solution, however, stabilization of the oligomers may require the additional association of the subunits with RNA. Binding to RNA would align N proteins to each other and increase their local concentration, thus stabilizing the inter-molecular interactions of the N-terminal arms and resulting in the stable, ring-shaped oligomers seen in SEC peak N1 (**Figure 6**). This model of RNA-stabilized oligomers provides an elegant molecular explanation for why N proteins can have an inherent tendency to multimerize without forming undesired, large oligomers in the absence of RNA.

**Figure 7:**
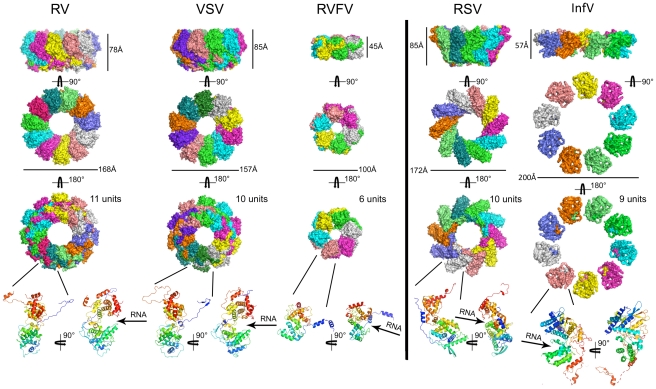
Gallery of crystal structures of N proteins from different negative strand RNA viruses. The structures shown are those of the N proteins from the rabies virus (RV; PDB code: 2GTT), the vesicular stomatitis virus (VSV; PDB code: 2GIC), the Rift Valley fever virus (RVFV; this work), the respiratory syncytial virus (RSV; PDB code: 2WJ8), and the influenza virus (InfV; PDB code: 2IQH). The model fro the influenza NP ring is derived from an EM reconstruction into which the crystal structure of the monomer was modeled (PDB code: 2WFS). The top three rows show different views of the ring structures in surface representation, in which each subunit is shown in a different color. The fourth row shows two views of the monomers in ribbon representation. The arrows indicate the cavity that binds the vRNA.

With only six subunits and a diameter of 100 Å, the RVFV N ring is the smallest one among the ring-shaped oligomers seen in crystal structures of N proteins from negative strand viruses (**Figure 7**). Although there are clearly common structural features in the oligomers, the mode of how the subunits interact with each other varies. In rabies virus (RV), vesicular stomatitis virus (VSV), respiratory syncytial virus (RSV) and influenza virus, extensions at both the N and C termini of the polypeptide are involved in organizing adjacent subunits into an ordered assembly [11], [12], [14]. By contrast, it is only the interaction of the N-terminal arm of RVFV N with the hydrophobic pocket of the neighboring subunit that mediates the contacts between adjacent subunits in oligomers. While this interaction appears sufficient to promote efficient protein polymerization, it leaves a significant degree of freedom at the level of lateral interactions. This plasticity is illustrated by the slightly different positions of the N-terminal arm on the core domain of the neighboring subunit seen in hexamers I and II in the crystals of the native and seleniated proteins (**Figure S5**). As a result, like the N proteins of RV and RSV, RVFV N can form rings with deformed shapes and a variable number of subunits (**Figure 6B**) and, although not yet observed, N may even have the capacity to form oligomers with a superhelical arrangement of the subunits.

Although EM of RVFV N-RNA complexes also showed ring-shaped oligomers (**Figure 6**), it is not clear whether rings are the building block of the native ribonucleocapsid. The RNPs of several Mononegavirales have a superhelical subunit arrangement, including those of RV [11], VSV [12], RSV [14], measles [9], [10] and mumps [13]. However, the RNPs of *Phleboviruses* do not assemble into a highly ordered structure, but rather into flexible filamentous assemblies [15], [23], [24], [25], [26]. In particular, EM images of RNPs from RVFV [15] and other *Bunyaviridae* [23], [24], [25] display an extended filament-like structure, but they do not rule out some degree of symmetry in the way the vRNA is packaged. While it thus remains uncertain whether the RVFV ribonucleocapsid is formed by stacked rings or a superhelical oligomer or even a mixture thereof, the flexibility in the interaction between adjacent subunits would allow great variability in the architecture of the ribonucleocapsid. Flexibility in the contacts between N subunits allows the assembly to readily adapt to distortions introduced by external constraints or signals within the infected cells, while maintaining the connectivity of the RNA.

The crystal structure of the hexamer reveals that several positively charged residues are clustered in a cleft that can accommodate a single molecule of RNA (**Figures 5** **and S8)**. This finding is in agreement with the proposal of Luo and collaborators that N RNA binding site is formed by two domains that contain a “(5H+3H)” structural motif [19]. Genomic RNA would thus run like a belt inside the ring and be completely concealed from the innate immune system of the host cell, in a manner similar to the ribonucleocapsid of the rabies virus [11]. Each N subunit can accommodate up to six bases, so that one turn of the RNA inside the hexameric ring would translate to∼36 bases. Our single-particle EM reconstruction of the hexameric RVFV N-RNA complex is consistent with the crystallographic hexamer of N, but it does not show the RNA inside the ring (**Figure 6D**). Considering the limited resolution of the EM density map of 25 Å, the small mass of 30 RNA bases and the negative charge of RNA, which would favor positive staining of the RNA, it is not surprising that the RNA is not visible in the EM map. By considering that the thickness of the hexameric N-RNA complex is about 45 Å (**Figure 6C**) and making the simplifying assumption that the entire nucleocapsid is formed by stacked hexamers, the ribonucleocapsid of the S segment of 1690 nt would span a total linear distance of about 0.25 µm. This value is consistent with the size of 0.27 µm of the ribonucleocapsid of Uukuniemi virus seen in EM images [27], whose genome is only slightly larger than that of RVFV.

Earlier studies showed that transcription and replication require not only the polymerase L but also the N protein [28], implying that naked vRNA cannot be transcribed [1]. While it has been established that the two proteins are positioned in close proximity to each other, as L is recruited to the vRNA through a panhandle structure [29] and N through a short region in the 5′ region of the ORF [30], how the two proteins interact with each other remains unclear. A recent study found a conserved region in the second domain of N consisting of helices α4, α5, α6 [31]. This domain may well play a role in the stacking of N subunits in the oligomer, but it could also mediate a transient interaction with L and promote a temporary release of N, thus liberating the RNA to become accessible for transcription by L. Additionally, helices α1, α12 and α13 are located at the periphery of the hexameric ring, and residues projecting from these helices are also likely to form a significant part of the L-binding surface on the ribonucleocapsid. Given the substantial contribution of the N-terminal arm to the buried interface (1456 Å^2^ out of a total of 1640 Å^2^ buried surface between two adjacent N subunits), it is conceivable that interactions of α1 of the N-terminal arm with L could lead to a local unwinding of the filament structure and the exposure of vRNA while avoiding complete disassembly of the ribonucleocapsid.

In conclusion, the structure of the hexamer formed by the RVFV N protein presented here shows that oligomerization is mediated by a flexible N-terminal arm, which binds a hydrophobic pocket in the adjacent subunit. The different hexamers seen in our crystals and the variability in the oligomeric state of N-RNA complexes seen in EM images demonstrate substantial flexibility in the interaction between subunits. Furthermore, comparison with a previous structure of the RVFV N protein suggests an elegant mechanism that allows the formation of stable N oligomers only in the presence of RNA. Finally, the nucleoprotein structure identifies potential sites that could be targeted for drug development. For instance, compounds blocking ribonucleocapsid assembly either by interfering with RNA binding or by trapping the N-terminal arm of N in a conformation that is not compatible with oligomerization, could serve as starting points to design specific antiviral molecules.

## Materials and Methods

### Cloning, mutagenesis, protein production and purification

cDNA corresponding to the RVFV N protein (strain Smithburn DQ380157.1) was cloned by recombination (Gateway, Invitrogen) into the pETG20A vector (kindly provided by Dr. Arie Geerlof), which adds a cleavable N-terminal thioredoxin-hexahistidine tag, and used to transform *E. coli* strain C41 (Avidis) carrying the pRARE plasmid (Novagen). Bacteria were grown in TB medium (Athena Enzyme) at 37°C to an OD_600nm_ of 0.5. Expression was induced with 0.5 mM IPTG, and bacteria were grown overnight at 17°C. Cells were pelleted, resuspended in 30 ml of lysis buffer (50 mM Tris, pH 8, 300 mM NaCl, 5 mM imidazole, 5% glycerol, 0.1% Triton X-100, 2 mM EDTA), frozen, and stored at -80°C.

N was purified at 4°C. A frozen pellet was melted on ice, sonicated, and the lysate was cleared by centrifugation at 20,000 rpm for 30 min. The protein was first purified by metal affinity chromatography using a 5 ml HisPrep column (GE Healthcare). The tag was then removed by cleavage with TEV protease, and the protein was further purified with a second metal affinity column followed by size exclusion chromatography (SEC) using a Superdex 200 column (GE Healthcare) in 10 mM HEPES, pH 7.5, 300 mM NaCl.

The R64D/K67D/K74D triple mutant was generated by first simultaneously introducing the R64D and K67D mutations followed by introducing the K74D mutation into the RVFV N cDNA using the QuickChange Site-Directed Mutagenis Kit (Agilent). The sequences of the primers used to introduce the point mutations were:

R64D/K67D forward: CTGGCTCTAACTgaTGGCAACgAcCCCCGGAGGATG,

R64D/K67D reverse: CATCCTCCGGGGgTcGTTGCCAtcAGTTAGAGCCAG,

K74D forward: CGGAGGATGATGATGgAcATGTCAAAAGAAGGC, and

K74D reverse: GCCTTCTTTTGACATgTcCATCATCATCCTCCG.

The complete coding region of each mutant was sequenced to confirm the desired modification. The triple mutant was expressed and purified analogous to the wild-type protein, and the expressed protein was verified by mass spectrometry.

Analytical SEC was performed on a KW 803 column (Shodex) using a High Pressure Liquid Chromatography Alliance 2695 system (Waters), and absorbance was measured at both 260 nm and 280 nm. The SEC column was calibrated with Kit LMW markers (GE Healthcare). The protein eluted in two peaks, with apparent molecular weights of∼300 kDa (N_1_) and 94 kDa (N_2_). The N_1_ peak was used for EM analysis of N-RNA complexes and the N_2_ peak for 3D crystallization screens of N.

### Characterization of N-RNA interactions by surface plasmon resonance spectroscopy

Binding affinities of wild-type and mutant N protein for ssRNA were determined using a ProteOn XPR36 instrument (Bio-Rad Laboratories, Inc). NeutrAvidin (Thermo Scientific) was amine-coupled to a carboxylated sensor surface (GLM sensor chip) to a final immobilized level of 6000 RU. To test non-specific binding by N, biotin-labeled ssRNA oligonucleotides with a non-relevant sequence from the dengue virus 5′ non-translated region (RNA_20_-3′biotin: GAGUUGUUAAUCUUUUUUUU-biotin; Sigma) were diluted to 10 nM in sodium acetate (pH 5.5) and injected for two minutes at a flow rate of 25 µl/min. Association and dissociation phases were measured for 240 sec and 600 sec, respectively. Measurements were performed in buffer containing 10 mM HEPES, pH 7.5, 300 mM NaCl, 0.005% NP-20. Data were analyzed in ProteOn Manager version 2.0.

### Crystallization

N protein in 10 mM HEPES, pH 7.5, 300 mM NaCl collected from fraction N_2_ was concentrated to 7.8 mg/ml, and 2 µl of protein solution was mixed with 2 µl of reservoir solution containing 200 mM MgNO_3_ and 17% (w/v) PEG 3350 for crystallization at 20°C using the hanging drop method. SDS-PAGE analysis of dissolved crystals confirmed that they contained full-length N protein. The crystals were flash-frozen in liquid nitrogen using 5% glycerol as cryo-protectant.

### Data collection, structure determination and refinement

The RVFV N protein crystallizes in space group P6 with unit cell parameters of a = b = 180.9 Å, c = 47.7 Å for the native protein and a = b = 175.5 Å, c = 47.4 Å for the seleniated protein. A native data set extending to 1.6 Å resolution and a Se-Met data set extending to 2.3 Å resolution were collected on beamline ID14–4 at the ESRF (Grenoble, France). The Se-Met data set was collected at the Se absorption edge. Data were processed using the program XDS [32]. Of a total of 33 Se sites for the three monomers in the asymmetric unit, the position of 27 sites were identified using the program SHELXD [33] to analyze anomalous data ranging from 10 to 2.3 Å. After initial phase calculation and modifications with the SHELX suite, a readily interpretable map was obtained with an overall figure of merit of 0.62. The program ARP/wARP [34] was used to generate an initial model, and a complete model for the three independent monomers was built using COOT [35]. Using this model, the native data set was subsequently solved by molecular replacement using the program Phaser [36]. The program REFMAC5 with the TLS option was used for crystallographic refinement [37]. The final models were assessed with PROCHECK [38]. Surface electrostatics were calculated using DELPHI [39]. Sequences were aligned using Muscle [40] and seaview [41]. Intermediate structures for the morphing were generated using LSQman [42]. Figures and movie were generated with the programs ENDscript, ESPript [43] and PyMOL (http://www.pymol.org).

### Sample preparation, electron microscopy and image processing

Samples were prepared by negative staining and cryo-negative staining with uranyl formate as described [44]. For specimens prepared by conventional negative staining, images were taken using Philips CM10 electron microscope equipped with a tungsten filament and operated at an acceleration voltage of 100 kV. Images were recorded on a 1 k×1 k Gatan CCD camera at a magnification of 52,000×using a defocus value of –1.5 µm. For cryo-negative staining specimens, images were recorded using a Tecnai F20 electron microscope (FEI), equipped with a field emission gun and operated at an acceleration voltage of 200 kV. Grids of cryo-negatively stained specimens, used to collect image pairs of specimens tilted to 50° and 0°, were loaded on an Oxford cryo-transfer holder and maintained at liquid nitrogen temperature during image acquisition. Images were taken at a magnification of 50,000×, with a defocus value of –2.0 µm for images of untilted specimens and –1.8 µm for specimens tilted to 50°. All images were recorded using low-dose procedures on Kodak film SO163 and developed for 12 min with full-strength Kodak D-19 developer at 20°C.

Electron micrographs were digitized with a SCAI scanner (Zeiss) using a step size of 7 µm, and 3×3 pixels were averaged to obtain a pixel size of 4.2 Å on the specimen level for cryo-negatively stained specimens. 3D reconstructions from the cryo-negatively stained preparations were calculated using the SPIDER software package [45]. 10,764 particle pairs were interactively selected from a total of 30 image pairs using WEB, the display program associated with SPIDER, and windowed into small images of 60×60 pixels. The particles from the images of the untilted specimens were classified over 10 cycles of *K* means classification and multi-reference alignment specifying 100 output classes. 3D density maps of individual classes were calculated with the corresponding particles selected from the images of the tilted specimen and using the back-projection, back-projection refinement, and angular refinement procedures implemented in SPIDER. The final 3D reconstruction of the hexameric N-RNA complex included 439 particles (399 particles from images of tilted specimens and 40 particles from images of untilted specimens) and its resolution was estimated by Fourier shell correlation (FSC) to be 25 Å according to the FSC = 0.5 criterion. The crystal structure of hexamer I formed by native RVFV N was first manually docked into the EM density map and then refined using the program UCSF Chimera [46].

## Supporting Information

Figure S1 Surface plasmon resonance spectroscopy analysis of RNA binding by RVFV N.A 20-nucleotides-long RNA was immobilized on a NeutrAvidin chip, and association and dissociation phases were measured for 240 sec and 600 sec, respectively, using the indicated concentrations of RVFV N. Data are representative of two independent experiments.
(TIF)

Figure S2 Hexamers in the crystals form tubes in the direction of the *c* axis. Top and side view of the tubes formed by hexamers I and II (same color code as in Figure 2) in the direction of the *c* axis. The six subunits in hexamer II are labeled A to F. The arrows indicate that the two tubes run in opposite directions.
(TIF)

Figure S3 Comparison of the two crystal structures of RVFV N. **(A)** Ribbon representation of the two crystal structures of an RVFV N subunit in the hexamer (PDB code: 3OU9) and as monomer (PDB code: 3LYF). Color code is the same as in Figure 3. **(B)** Side and top view of a superimposition of the two crystal structures based on the core domain. The hexamer structure is shown in orange (PDb code: 3OU9) and the monomer structure in purple (PDB code: (PDB code: 3LYF [15]). The rmsd between the backbone atoms of the core domains is∼0.7 Å. The U-shaped arrow indicates the movement of the N-terminal arm.
(TIF)

Figure S4 Multiple sequence alignment of N proteins from the *Phlebovirus* family. Invariant residues are shown in white with red background, conserved residues are shown in red with white background, and variable residues are shown in black with white background. The secondary structure elements are indicated above the alignment with the same color code used in Figure 3. The sequence alignment was generated with ClustalW, and secondary structure was assigned with ESPript. The sequences and their database accession numbers are: Rift Valley fever virus (GI 9632367), Phlebovirus sp. Be Ar 371637 (GI 146336853), Phlebovirus sp. VP-161A (GI 146336850), Phlebovirus sp. PAN 483391 (GI 146336925) Phlebovirus sp. Pa Ar 2381 (GI 146336916), Punta Toro virus (GI 146336898), Phlebovirus sp. GML 902878 (GI 146336904), Phlebovirus sp. VP-366G (GI 146336907), Phlebovirus sp. Co Ar 171616 (GI 146336901), Buenaventura virus (GI 146336910), Sandfly fever sicilian virus (GI 146336868), Corfou virus (GI 146336856), Massilia virus (GI 208610196), Sandfly fever Naples virus (GI 146336886), and Uukuniemi virus (GI 38371708).
(TIF)

Figure S5 Comparison of hexamers formed by native and seleniated RVFV N. **(A)** The left panel shows hexamers I and II formed by native protein in pink and cyan, respectively, and the right panel shows hexamers I and II formed by seleniated protein in marine and yellow, respectively. Hexamer I formed by seleniated N has a different organization from all the other hexamers. **(B)** Superimposition of hexamer I formed by native N (pink) with hexamers II formed by native and seleniated N (cyan and yellow), showing that the subunits in these rings have an identical arrangement. **(C)** Superimposition of hexamer I formed by native N (pink) with hexamer I formed by seleniated N (marine), revealing an 11° rotation between the planes of the two rings.
(TIF)

Figure S6 Structural variability in the N protein and in N-N interactions. **(A)** N-N interaction in hexamer I formed by the native protein. In panels A to C, the subunit shown in grey surface representation is fixed, with the hydrophobic groove shown in color, and the interacting subunit is shown in wire mesh. **(B)** N-N interaction in hexamer I formed by the seleniated protein. **(C)** Comparison of the N-N interactions shown in panels A and B. The superimposition reveals a shift of the protein core with respect to the N-terminal arm (lateral slippage). **(D)** Comparison of the relative position of the N-terminal arm in hexamer I formed by the native protein (top panel) and hexamer I formed by the seleniated protein (bottom panel). In panels D to G, the native proteins are shown in cyan and yellow and the seleniated proteins in marine and brown. **(E)** Comparison of the angle between two subunits in hexamer I formed by the native protein (top panel) and hexamer I formed by the seleniated protein (bottom panel), showing that the angle is identical. **(F)** Comparison of the relative position of two subunits in hexamer I formed by the native protein (left panel) and hexamer I formed by the seleniated protein (right panel), showing a deviation of 11° between the two monomers. **(G)** The arrangement of the cores of two subunits shows a lateral slippage of 2.3 Å.
(TIF)

Figure S7 The R64D/K67D/K74D triple mutant fails to bind RNA. **(A)** Elution profile of the R64D/K67D/K74D triple N mutant from a S200 size exclusion column. The blue line shows the absorbance at 280 nm. The inset shows a 12.5% SDS-PAGE gel of the elution fractions of the peak, revealing a protein band at 27 kDa. **(B)** Surface plasmon resonance profile for binding of a 20-nucleotides-long RNA by wild-type N (blue line) and the triple mutant (red line). Association and dissociation phases were measured for 100 sec and 500 sec, respectively.
(TIF)

Figure S8 Modeling of an RNA molecule into the basic cleft of an RVFV N dimer. The RNA molecule was positioned based on the RNA seen in the structure of the N protein from RV (PDB code: 2GTT; [11]) with some manual adjustments in order to fit the RNA molecule into the cavity. The RNA is colored according to the atoms, with carbon in white, oxygen in red, phosphate in orange, and nitrogen in blue. Positively charged residues that were substituted in the triple mutant are shown as sticks and labeled.
(TIF)

Figure S9 Class averages of cryo-negatively stained N-RNA oligomers. The 100 class averages, obtained from the classification of 10,764 particles, are arranged according to particle number such that the upper-left panel shows the average with the most particles and the lower-right panel shows the average with the least particles. The side length of the individual panels is 24 nm.
(TIF)

Figure S10 Fourier shell correlation (FSC) curve of the single-particle EM reconstruction of the hexameric N-RNA complex. The actual FSC curve (dashed line) and a smoothened representation (continuous line), suggesting that the density map has a resolution of 25 Å according to the FSC = 0.5 criterion.
(TIF)

Figure S11 Intra-molecular interaction of the N-terminal arm with its own core domain. **(A)** Surface representation of the N protein in monomeric form (PDB code: 3LYF; [15]). The surface is shown transparent and in purple except the hydrophobic residues that interact with the N-terminal arm, which are shown in green. The Cα backbone of N is shown in red and the side chains of the residues of the N-terminal arm that interact with the hydrophobic groove in the core domain are shown as yellow sticks. **(B)** Amino acid sequence of the RVFV N polypeptide, showing above the secondary structure elements derived from the crystal structure. Below the sequence, residues are labeled that are involved in intra-subunit interactions. Yellow dots indicate residues of the N-terminal arm interacting with the core domain and green bars indicate residues of the core domain interacting with the N-terminal arm in the monomer (PDB: 3LYF). For reference, residues involved in intermolecular interactions with the N-terminal arm of an adjacent molecule are indicated by an orange dot below the sequence (hexameric structure).
(TIF)

Video S1 Transition of the N-terminal arm between the positions seen in the two crystal structures.The crystal structures of monomeric N (PDB codes: 3LYF; [15]) and hexameric N (PDB codes: 3OU9) were overlayed based on the core domain, and the movement of the N-terminal arm between the positions seen in the two crystal structures was simulated using the program LSQMAN. The N-terminal arm is shown in red, the globular core domain in brown and green, and the C terminus in blue.
(MOV)
