# Supplementary figures and images for: The Hexamer Structure of the Rift Valley Fever Virus Nucleoprotein Suggests a Mechanism for its Assembly into Ribonucleoprotein Complexes
Source: PLoS Pathog. 2011 May 12;7(5):e1002030. doi: 10.1371/journal.ppat.1002030 (PMC3093367; doi:10.1371/journal.ppat.1002030)

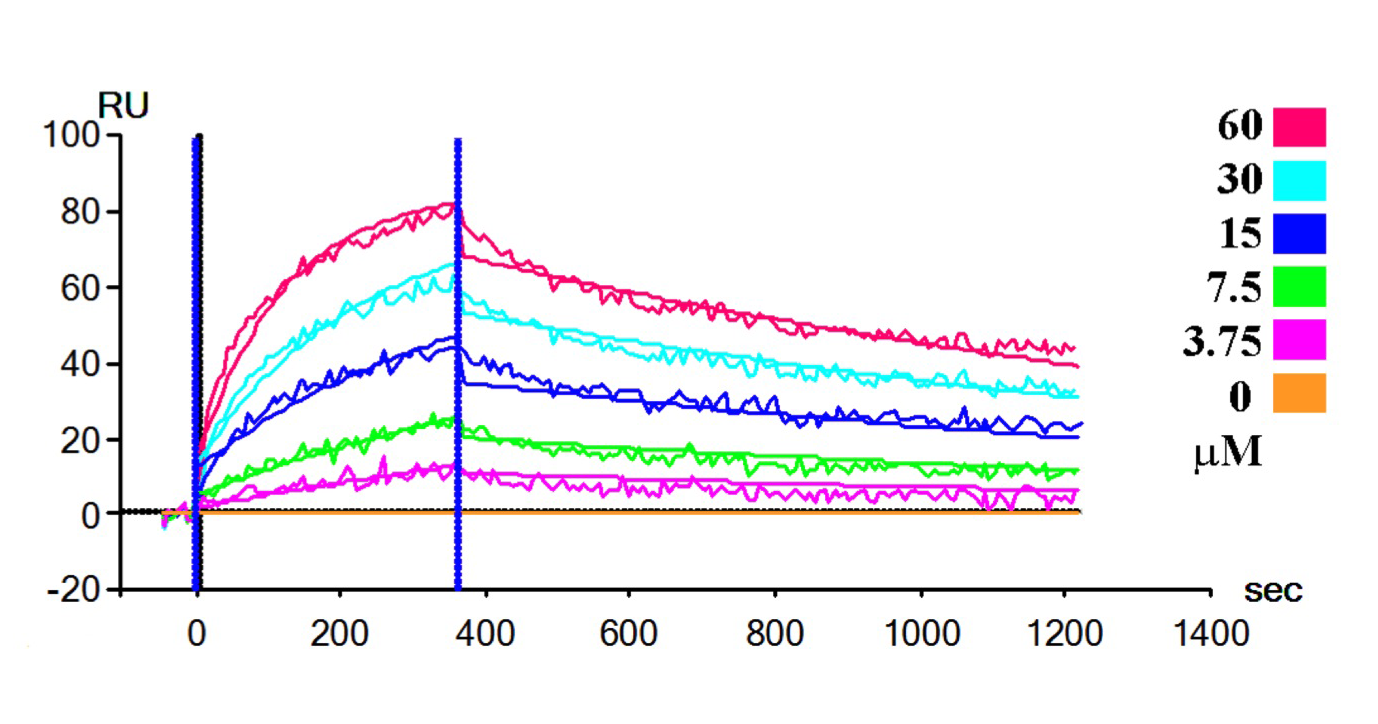

Supplement: Figure S1 — Surface plasmon resonance spectroscopy analysis of RNA binding by RVFV N.A 20-nucleotides-long RNA was immobilized on a NeutrAvidin chip, and association and dissociation phases were measured for 240 sec and 600 sec, respectively, using the indicated concentrations of RVFV N. Data are representative of two independent experiments. (TIF) [file ppat.1002030.s001.tif]

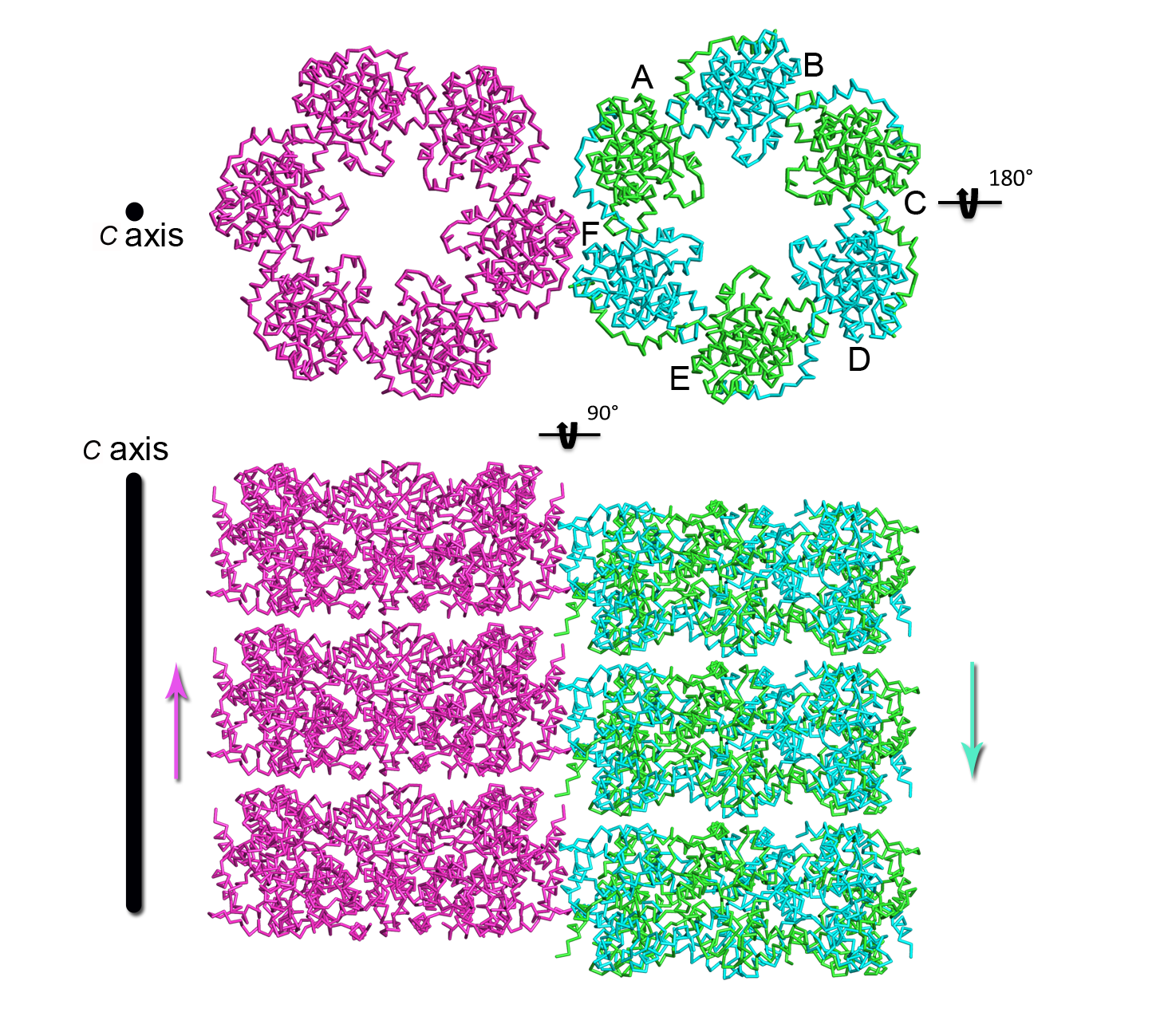

Supplement: Figure S2 — Hexamers in the crystals form tubes in the direction of the c axis. Top and side view of the tubes formed by hexamers I and II (same color code as in Figure 2) in the direction of the c axis. The six subunits in hexamer II are labeled A to F. The arrows indicate that the two tubes run in opposite directions. (TIF) [file ppat.1002030.s002.tif]

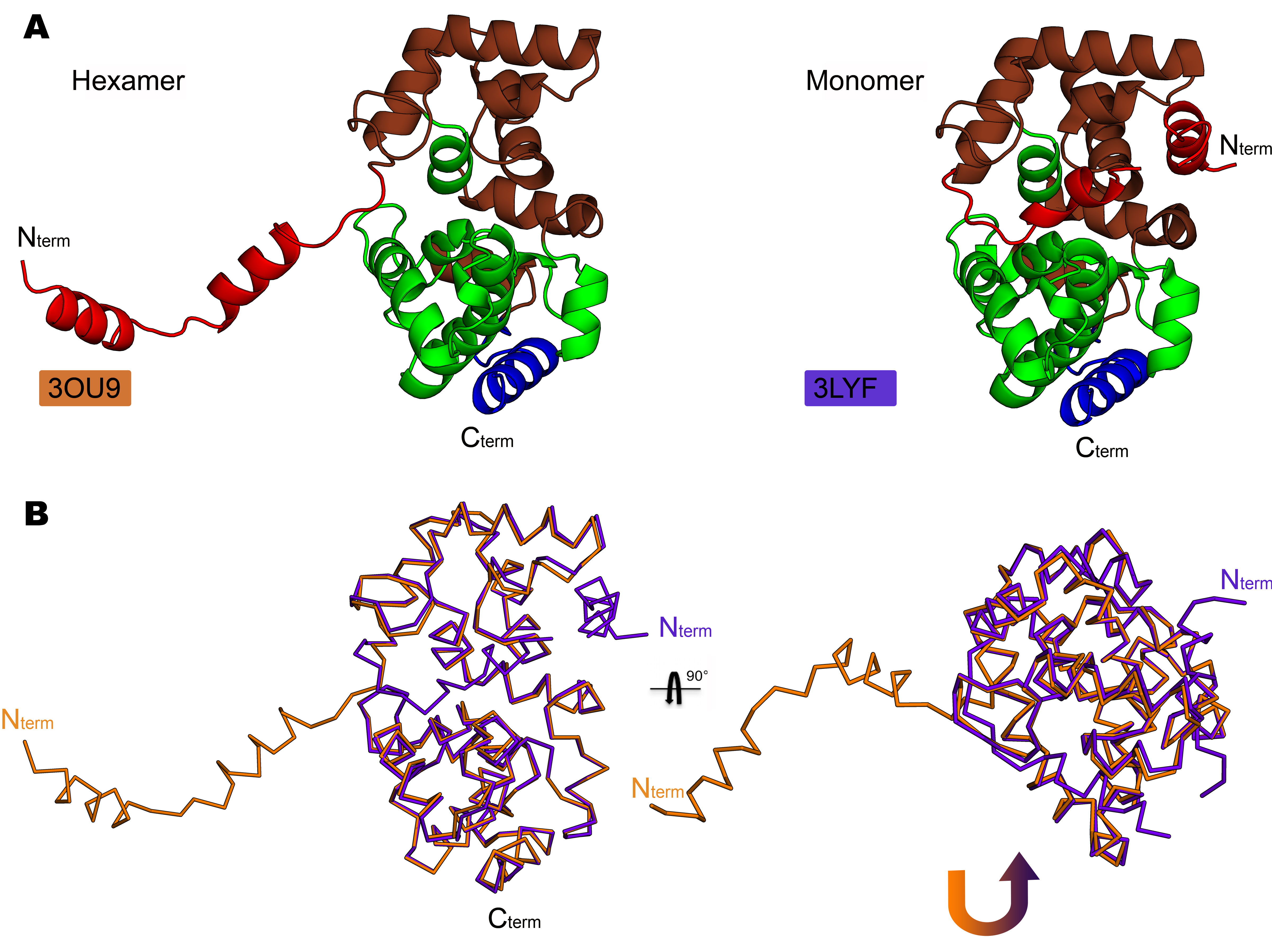

Supplement: Figure S3 — Comparison of the two crystal structures of RVFV N. (A) Ribbon representation of the two crystal structures of an RVFV N subunit in the hexamer (PDB code: 3OU9) and as monomer (PDB code: 3LYF). Color code is the same as in Figure 3. (B) Side and top view of a superimposition of the two crystal structures based on the core domain. The hexamer structure is shown in orange (PDb code: 3OU9) and the monomer structure in purple (PDB code: (PDB code: 3LYF [15]). The rmsd between the backbone atoms of the core domains is∼0.7 Å. The U-shaped arrow indicates the movement of the N-terminal arm. (TIF) [file ppat.1002030.s003.tif]

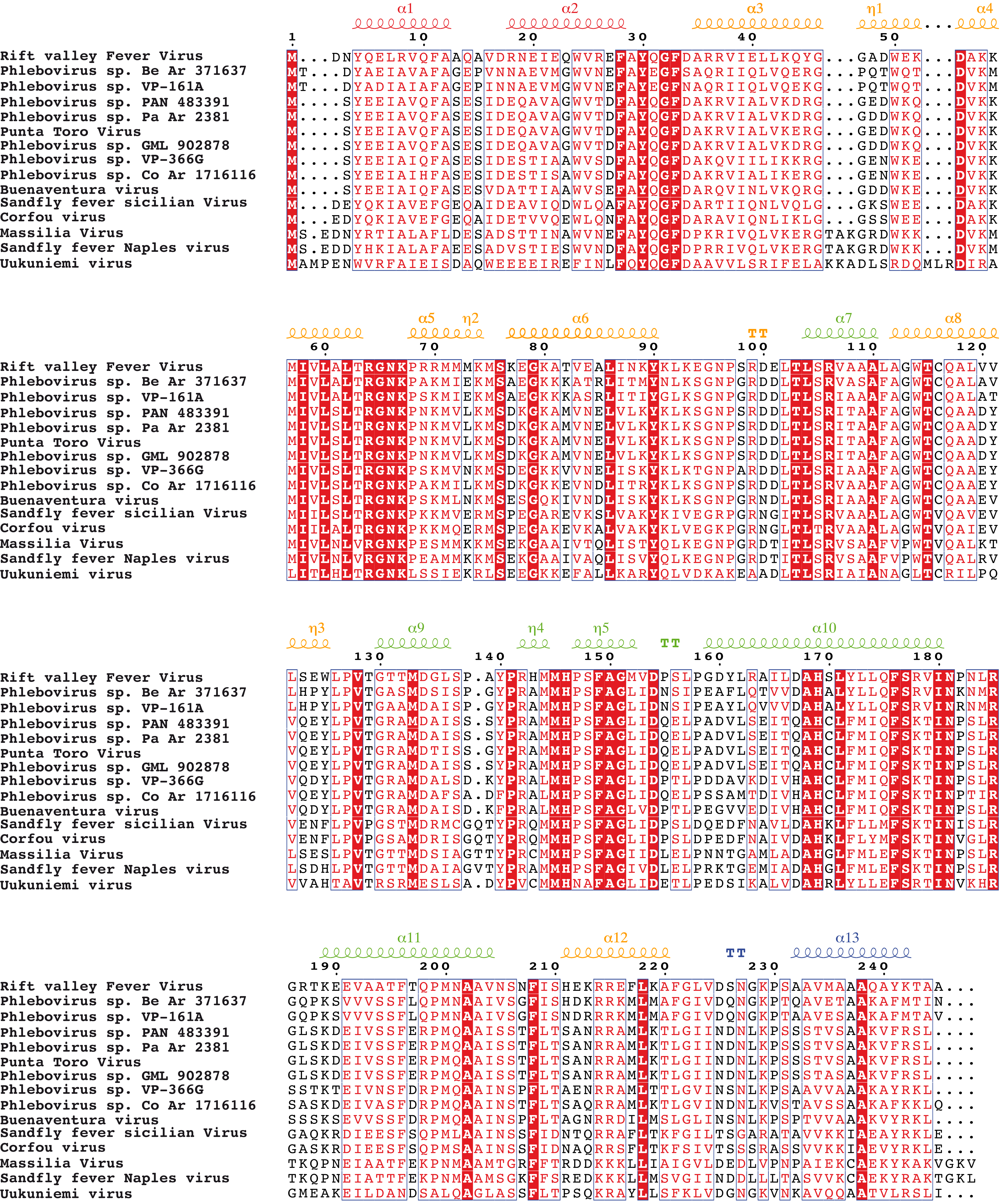

Supplement: Figure S4 — Multiple sequence alignment of N proteins from the Phlebovirus family. Invariant residues are shown in white with red background, conserved residues are shown in red with white background, and variable residues are shown in black with white background. The secondary structure elements are indicated above the alignment with the same color code used in Figure 3. The sequence alignment was generated with ClustalW, and secondary structure was assigned with ESPript. The sequences and their database accession numbers are: Rift Valley fever virus (GI 9632367), Phlebovirus sp. Be Ar 371637 (GI 146336853), Phlebovirus sp. VP-161A (GI 146336850), Phlebovirus sp. PAN 483391 (GI 146336925) Phlebovirus sp. Pa Ar 2381 (GI 146336916), Punta Toro virus (GI 146336898), Phlebovirus sp. GML 902878 (GI 146336904), Phlebovirus sp. VP-366G (GI 146336907), Phlebovirus sp. Co Ar 171616 (GI 146336901), Buenaventura virus (GI 146336910), Sandfly fever sicilian virus (GI 146336868), Corfou virus (GI 146336856), Massilia virus (GI 208610196), Sandfly fever Naples virus (GI 146336886), and Uukuniemi virus (GI 38371708). (TIF) [file ppat.1002030.s004.tif]

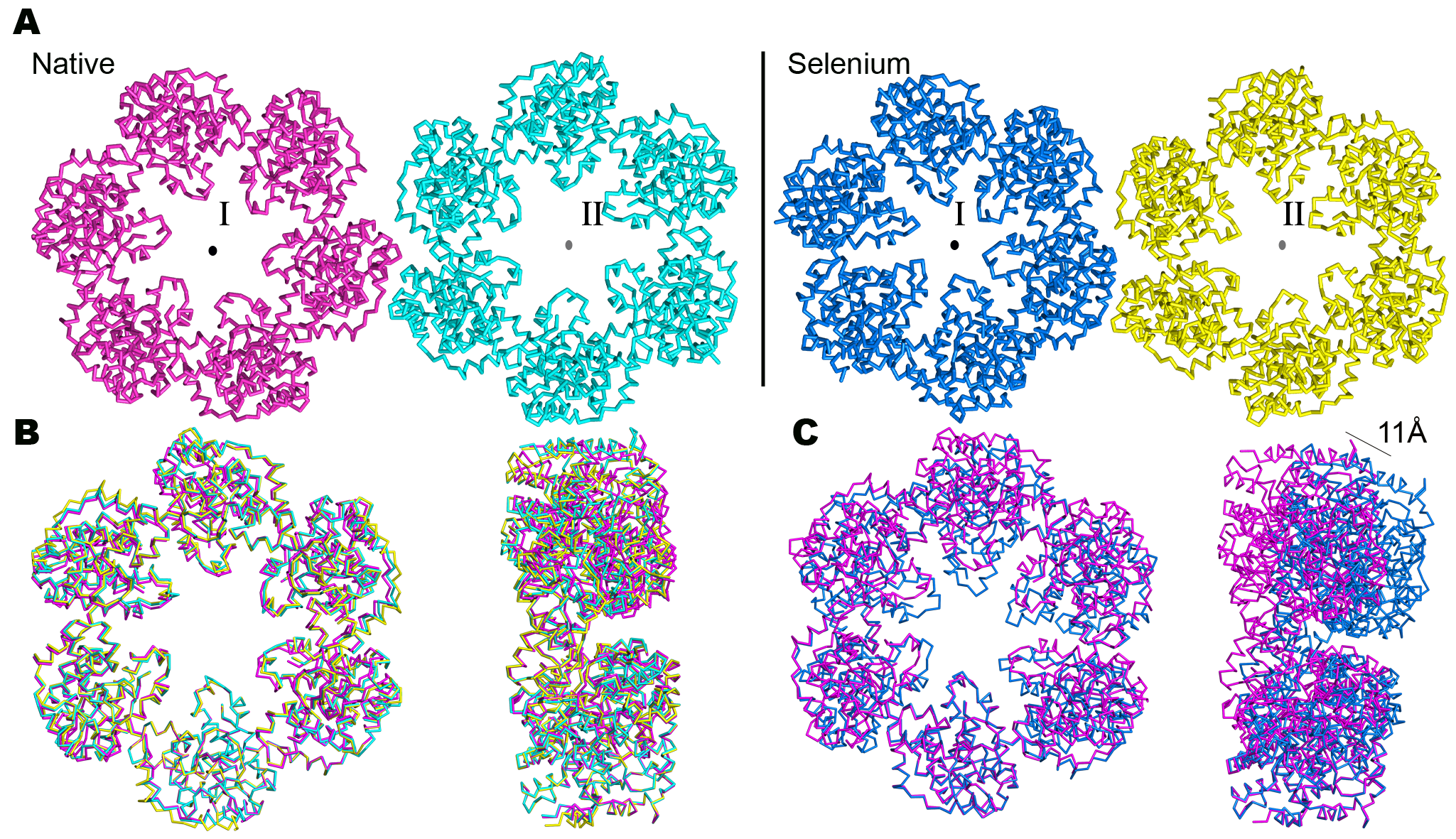

Supplement: Figure S5 — Comparison of hexamers formed by native and seleniated RVFV N. (A) The left panel shows hexamers I and II formed by native protein in pink and cyan, respectively, and the right panel shows hexamers I and II formed by seleniated protein in marine and yellow, respectively. Hexamer I formed by seleniated N has a different organization from all the other hexamers. (B) Superimposition of hexamer I formed by native N (pink) with hexamers II formed by native and seleniated N (cyan and yellow), showing that the subunits in these rings have an identical arrangement. (C) Superimposition of hexamer I formed by native N (pink) with hexamer I formed by seleniated N (marine), revealing an 11° rotation between the planes of the two rings. (TIF) [file ppat.1002030.s005.tif]

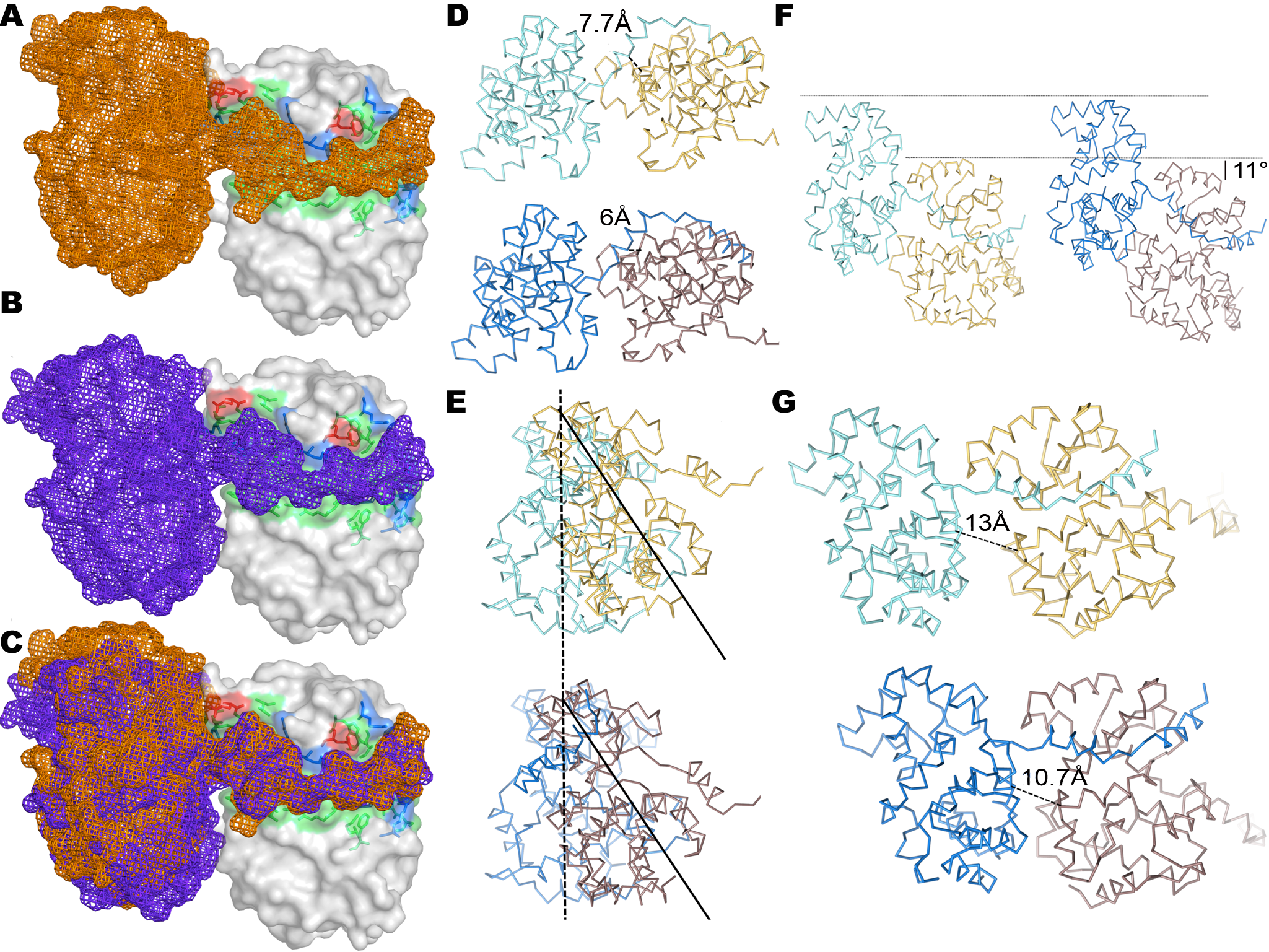

Supplement: Figure S6 — Structural variability in the N protein and in N-N interactions. (A) N-N interaction in hexamer I formed by the native protein. In panels A to C, the subunit shown in grey surface representation is fixed, with the hydrophobic groove shown in color, and the interacting subunit is shown in wire mesh. (B) N-N interaction in hexamer I formed by the seleniated protein. (C) Comparison of the N-N interactions shown in panels A and B. The superimposition reveals a shift of the protein core with respect to the N-terminal arm (lateral slippage). (D) Comparison of the relative position of the N-terminal arm in hexamer I formed by the native protein (top panel) and hexamer I formed by the seleniated protein (bottom panel). In panels D to G, the native proteins are shown in cyan and yellow and the seleniated proteins in marine and brown. (E) Comparison of the angle between two subunits in hexamer I formed by the native protein (top panel) and hexamer I formed by the seleniated protein (bottom panel), showing that the angle is identical. (F) Comparison of the relative position of two subunits in hexamer I formed by the native protein (left panel) and hexamer I formed by the seleniated protein (right panel), showing a deviation of 11° between the two monomers. (G) The arrangement of the cores of two subunits shows a lateral slippage of 2.3 Å. (TIF) [file ppat.1002030.s006.tif]

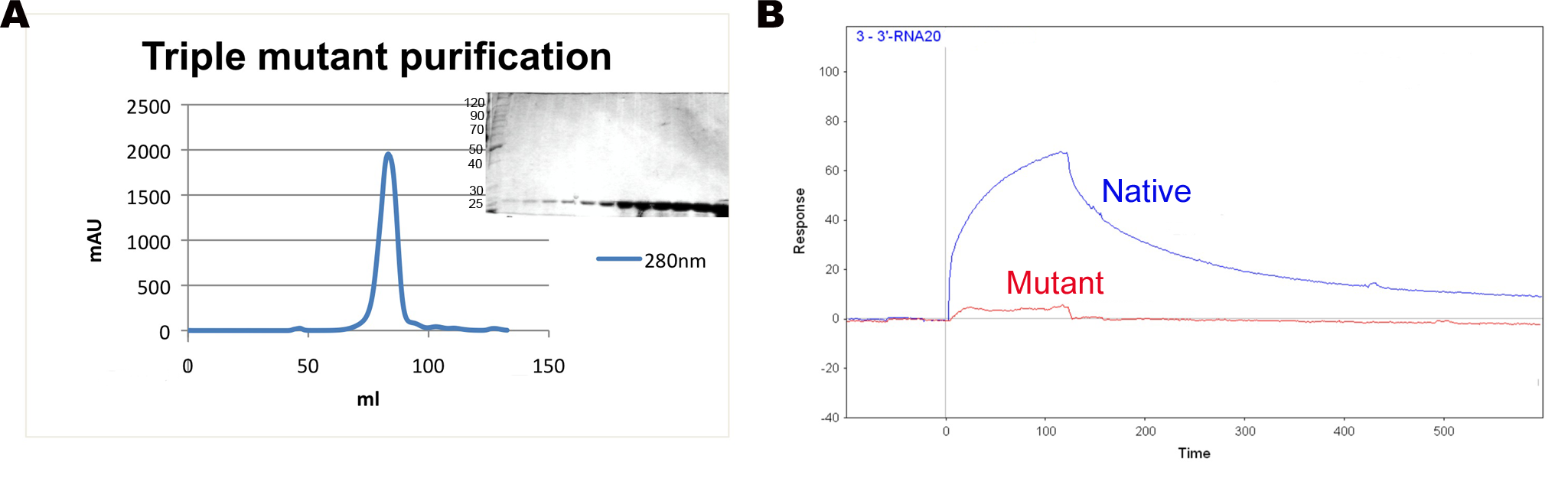

Supplement: Figure S7 — The R64D/K67D/K74D triple mutant fails to bind RNA. (A) Elution profile of the R64D/K67D/K74D triple N mutant from a S200 size exclusion column. The blue line shows the absorbance at 280 nm. The inset shows a 12.5% SDS-PAGE gel of the elution fractions of the peak, revealing a protein band at 27 kDa. (B) Surface plasmon resonance profile for binding of a 20-nucleotides-long RNA by wild-type N (blue line) and the triple mutant (red line). Association and dissociation phases were measured for 100 sec and 500 sec, respectively. (TIF) [file ppat.1002030.s007.tif]

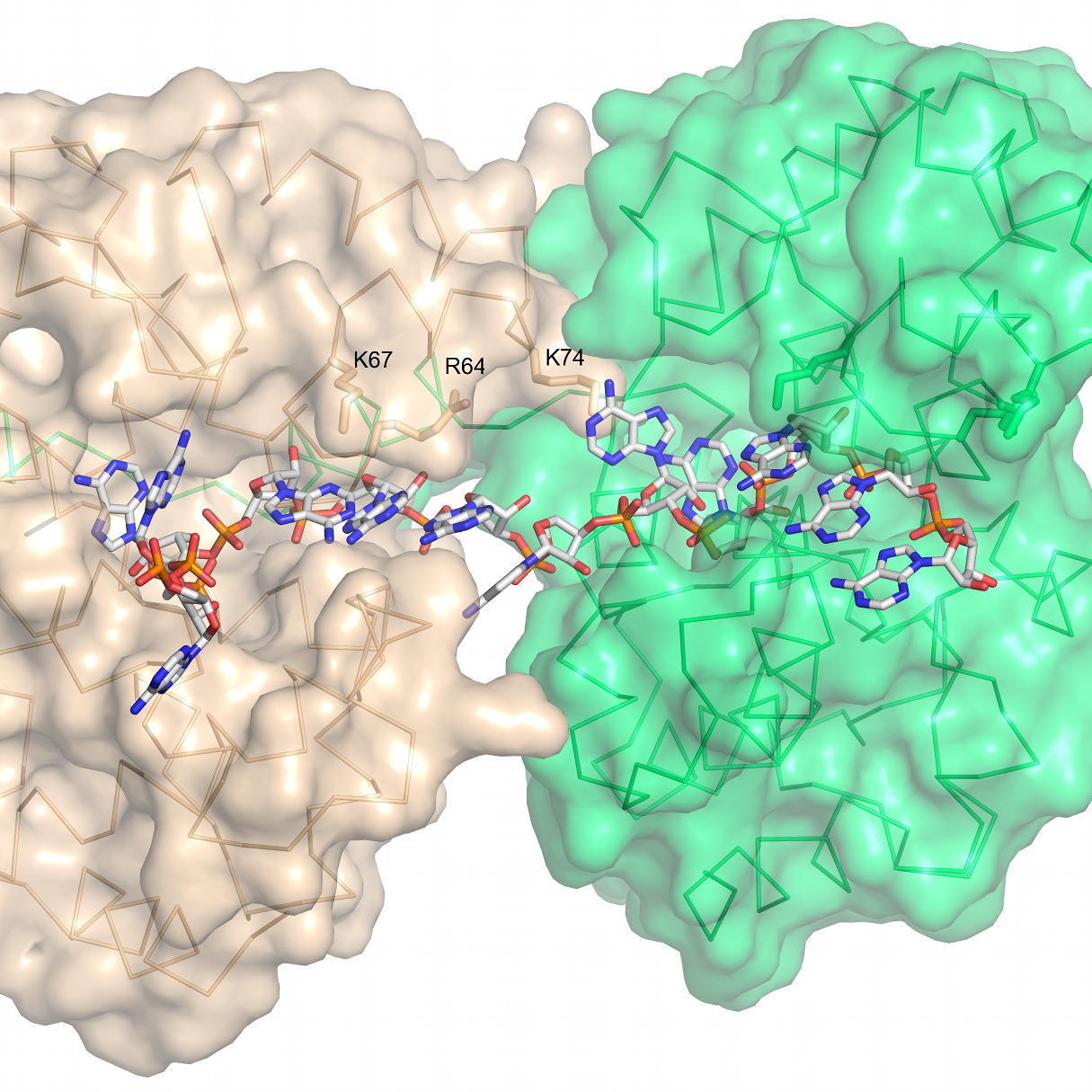

Supplement: Figure S8 — Modeling of an RNA molecule into the basic cleft of an RVFV N dimer. The RNA molecule was positioned based on the RNA seen in the structure of the N protein from RV (PDB code: 2GTT; [11]) with some manual adjustments in order to fit the RNA molecule into the cavity. The RNA is colored according to the atoms, with carbon in white, oxygen in red, phosphate in orange, and nitrogen in blue. Positively charged residues that were substituted in the triple mutant are shown as sticks and labeled. (TIF) [file ppat.1002030.s008.tif]

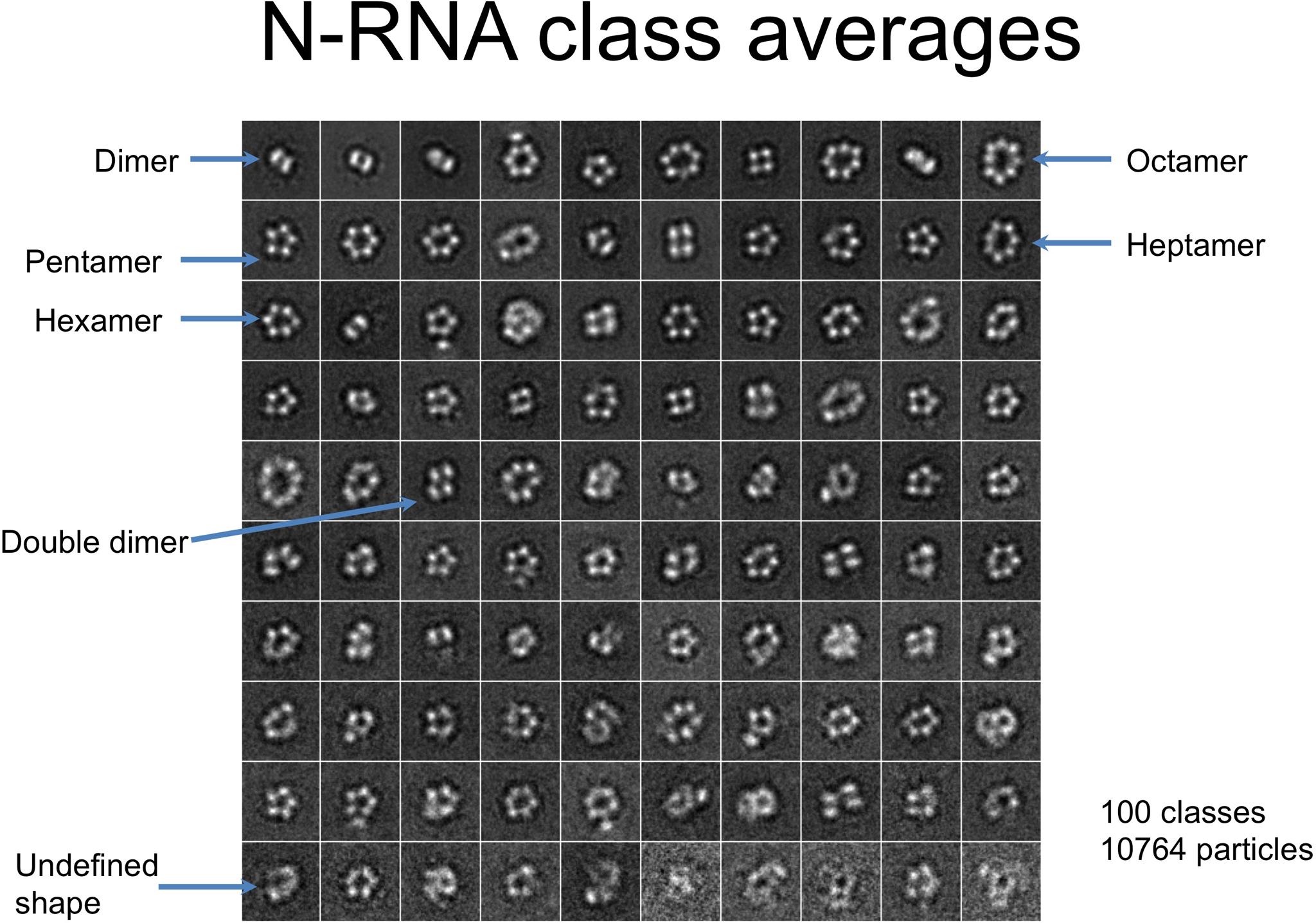

Supplement: Figure S9 — Class averages of cryo-negatively stained N-RNA oligomers. The 100 class averages, obtained from the classification of 10,764 particles, are arranged according to particle number such that the upper-left panel shows the average with the most particles and the lower-right panel shows the average with the least particles. The side length of the individual panels is 24 nm. (TIF) [file ppat.1002030.s009.tif]

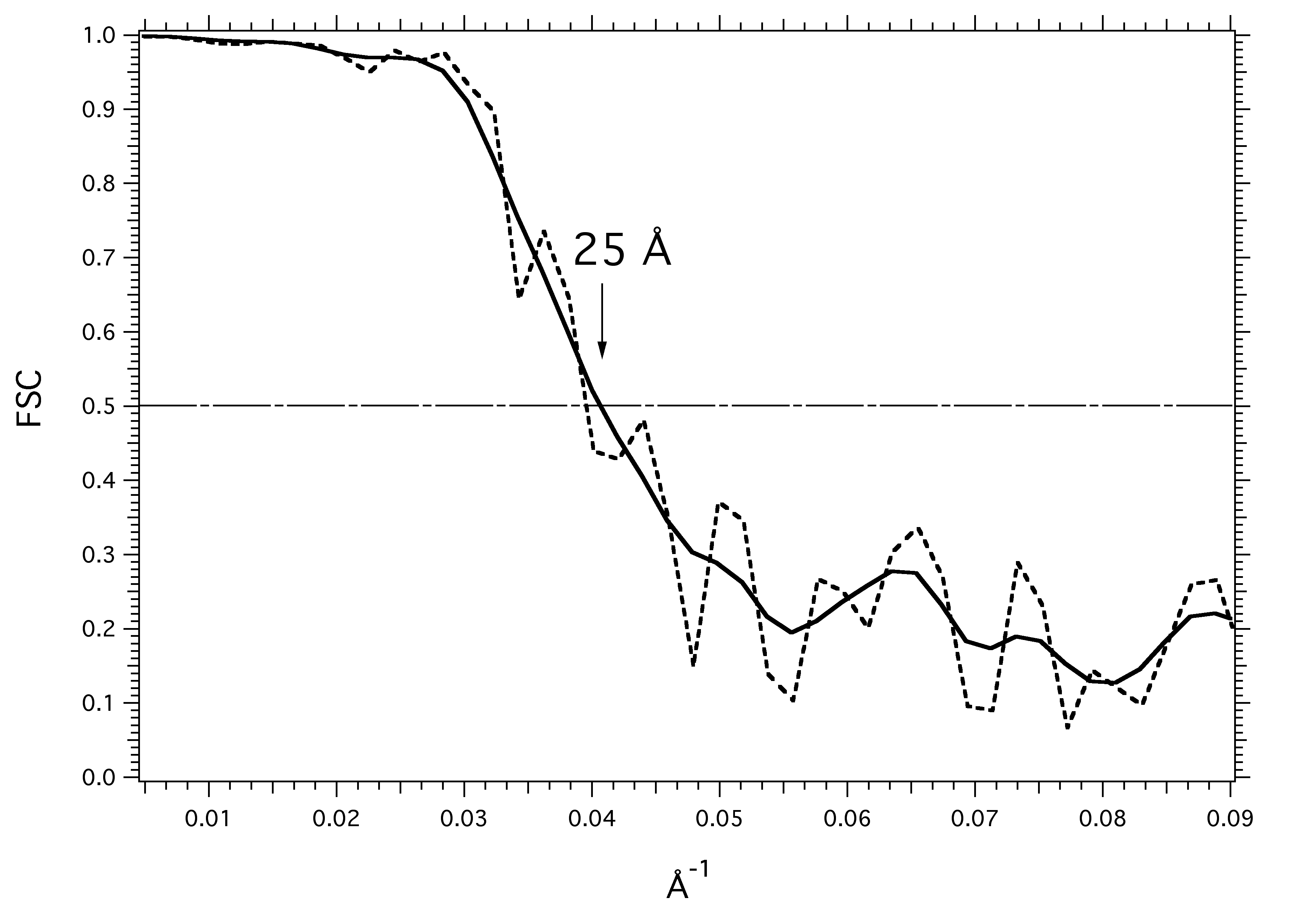

Supplement: Figure S10 — Fourier shell correlation (FSC) curve of the single-particle EM reconstruction of the hexameric N-RNA complex. The actual FSC curve (dashed line) and a smoothened representation (continuous line), suggesting that the density map has a resolution of 25 Å according to the FSC = 0.5 criterion. (TIF) [file ppat.1002030.s010.tif]

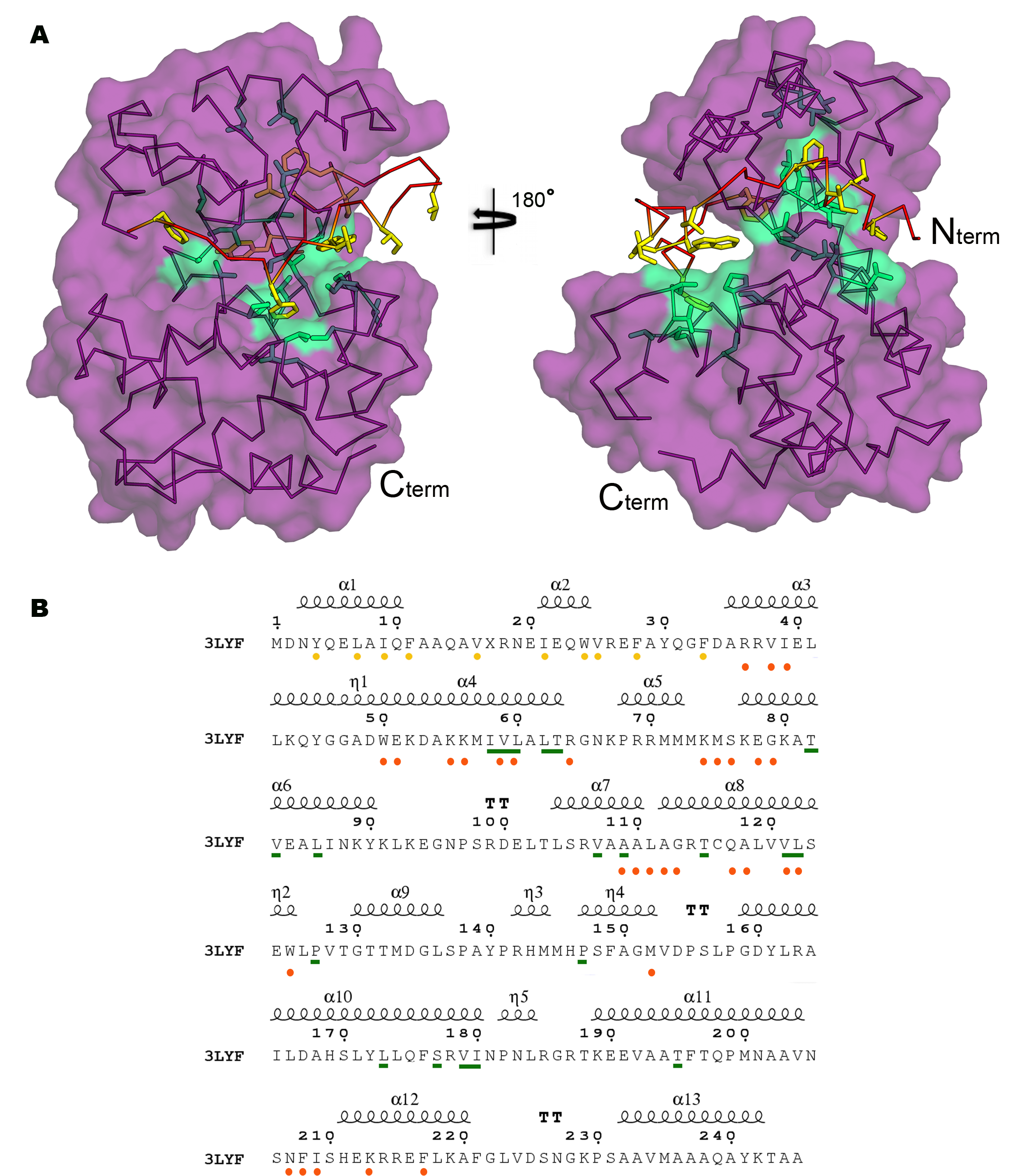

Supplement: Figure S11 — Intra-molecular interaction of the N-terminal arm with its own core domain. (A) Surface representation of the N protein in monomeric form (PDB code: 3LYF; [15]). The surface is shown transparent and in purple except the hydrophobic residues that interact with the N-terminal arm, which are shown in green. The Cα backbone of N is shown in red and the side chains of the residues of the N-terminal arm that interact with the hydrophobic groove in the core domain are shown as yellow sticks. (B) Amino acid sequence of the RVFV N polypeptide, showing above the secondary structure elements derived from the crystal structure. Below the sequence, residues are labeled that are involved in intra-subunit interactions. Yellow dots indicate residues of the N-terminal arm interacting with the core domain and green bars indicate residues of the core domain interacting with the N-terminal arm in the monomer (PDB: 3LYF). For reference, residues involved in intermolecular interactions with the N-terminal arm of an adjacent molecule are indicated by an orange dot below the sequence (hexameric structure). (TIF) [file ppat.1002030.s011.tif]
